# Complete Genome Sequences of Paenibacillus larvae Phages BN12, Dragolir, Kiel007, Leyra, Likha, Pagassa, PBL1c, and Tadhana

**DOI:** 10.1128/genomeA.01602-17

**Published:** 2018-06-14

**Authors:** Jamison K. Walker, Bryan D. Merrill, Jordan A. Berg, Aziza Dhalai, Douglas W. Dingman, Chris P. Fajardo, Kiel Graves, Hunter L. Hill, Jared A. Hilton, Cameron Imahara, Bradley K. Knabe, James Mangohig, Josh Monk, Heejin Mun, Ashley M. Payne, Alicia Salisbury, Casey Stamereilers, Kathie Velez, Andy T. Ward, Donald P. Breakwell, Julianne H. Grose, Sandra Hope, Philippos K. Tsourkas

**Affiliations:** a Department of Microbiology and Molecular Biology, Brigham Young University, Provo, Utah, USA; b School of Life Sciences, University of Nevada, Las Vegas, Nevada, USA; c Department of Entomology, Connecticut Agricultural Experiment Station, New Haven, Connecticut, USA

## Abstract

We present here the complete genomes of eight phages that infect Paenibacillus larvae, the causative agent of American foulbrood in honeybees. Phage PBL1c was originally isolated in 1984 from a P. larvae lysogen, while the remaining phages were isolated in 2014 from bee debris, honeycomb, and lysogens from three states in the USA.

## GENOME ANNOUNCEMENT

The Gram-positive bacterium Paenibacillus larvae is the causative agent of American foulbrood, currently the most destructive bacterial disease affecting the honeybee, Apis mellifera (1). With the rise of antibiotic-resistant strains of P. larvae (2), there is growing interest in phages that infect this pathogen. The first P. larvae phages were isolated in the 1950s (3), and the first complete P. larvae genome was published in 2013 (4). There are currently 18 complete P. larvae phage genomes in the literature (4–7). Here, we present eight complete P. larvae phage genomes obtained from samples across the United States. The phages’ GenBank accession numbers, isolation sources, geographical provenance, and assembly results are shown in Table 1.

**TABLE 1:** P. larvae phages, GenBank accession numbers, and genome assembly results

Phage name	GenBank accession no.	Isolation source	Location	Genome length (bp)	GC content (%)
BN12	MG727695	Bee debris	Cedar City, Utah, USA	39,485	42.6
Dragolir	MG727697	Bee debris	Wisconsin, USA	41,131	44
Kiel007	MG727696	Bee debris	Salt Lake City, Utah, USA	37,985	41.8
Leyra	MG727701	Bee debris	Idaho, USA	42,276	41.4
Likha	MG727702	Honeycomb	American Fork, Utah, USA	39,778	41.3
Pagassa	MG727699	P. larvae lysogen	Provo, Utah, USA	40,035	42
PBL1c	MG727698	P. larvae lysogen	Iowa City, Iowa, USA	40,611	41.2
Tadhana	MG727700	P. larvae lysogen	Provo, Utah, USA	37,880	42.1

Phage PBL1c was isolated from a lysogen in 1984 by Dingman et al. (8) but was not sequenced until 2018 at Brigham Young University (BYU). The remaining seven phages were isolated over the period 2014 to 2016 from samples from the USA states of Utah, Idaho, and Wisconsin (Table 1) as part of the Phage Hunters course at BYU.

The phages were isolated from bee debris, honeycomb, and lysogens and amplified in P. larvae field isolates. Phage genomic DNA was isolated from high-titer lysates using Norgen phage DNA isolation kits (Norgen Biotek, Thorold, ON, Canada). Phage genomes were sequenced in the BYU DNA Sequencing Center using the Illumina HiSeq 2500 platform (Illumina, Hayward, CA, USA) and were assembled using Geneious 8 software (Biomatters Inc., Newark, NJ, USA).

All nine phages are members of the family Siphoviridae with linear double-stranded DNA genomes. The DNA packaging strategy was identified as “cohesive ends with 3′ overhangs,” as explained in references 9 and 10. The overhangs were identified by sequence similarity with previously published phages (3–7). The overhangs are “CGACTGCCC” for phages BN12, Kiel007, Leyra, Likha, Pagassa, PBL1c, and Tadhana, and “CGACGGACC” for phage Dragolir. The genomes were rearranged by setting the first base of the genome to be the base immediately after the 3′ overhang.

Genome length is in the 37 kb to 42 kb range, and the G+C content was in the 41 to 44% range, consistent with 3′ cohesive ends for P. larvae phages (11). Preliminary analysis shows that phages Pagassa and Tadhana are closely related to each other, with the other phages slightly more distant; phage Dragolir was shown to be an outlier. All eight phages encode a large terminase, a major tail protein, two tail assembly proteins, a tail tape measure protein, and an *N*-acetylmuramoyl-l-alanine amidase, among others. The tail assembly proteins appear to have a programmed translational frameshift similar to the G and G-T genes of phage lambda (12, 13), located in the 3′ region of gp12 (the upstream tail assembly protein). We tentatively identified the heptanucleotide slippery sequence as “AAAAAAG” in phages BN12, Kiel007, Likha, Leyra, Pagassa, PBL1c, and Tadhana, and possibly “AAAAAAC” in phage Dragolir. Future studies will investigate this and other features of P. larvae phage genomes and also provide a detailed comparative genomic analysis of these and other P. larvae phages.

### Accession number(s).

The genome sequences of the P. larvae phages reported here have been deposited in GenBank under the accession numbers listed in Table 1.
